# Anion homeostasis is important for non-lytic release of BK polyomavirus from infected cells

**DOI:** 10.1098/rsob.150041

**Published:** 2015-08-05

**Authors:** Gareth L. Evans, Laura G. Caller, Victoria Foster, Colin M. Crump

**Affiliations:** Division of Virology, Department of Pathology, University of Cambridge, Tennis Court Road, Cambridge CB2 1QP, UK

**Keywords:** polyomavirus, BKPyV, DIDS, anion homeostasis, virus release

## Abstract

BK polyomavirus (BKPyV) is a member of a family of potentially oncogenic viruses, whose reactivation can cause severe pathological conditions in transplant patients, leading to graft rejection. As with many non-enveloped viruses, it is assumed that virus release occurs through lysis of the host cell. We now show the first evidence for a non-lytic release pathway for BKPyV and that this pathway can be blocked by the anion channel inhibitor DIDS. Our data show a dose-dependent effect of DIDS on the release of BKPyV virions. We also observed an accumulation of viral capsids in large LAMP-1-positive acidic organelles within the cytoplasm of cells upon DIDS treatment, suggesting potential late endosome or lysosome-related compartments are involved in non-lytic BKPyV release. These data highlight a novel mechanism by which polyomaviruses can be released from infected cells in an active and non-lytic manner, and that anion homeostasis regulation is important in this pathway.

## 1. Introduction

BK polyomavirus (BKPyV) was identified in 1971 at a similar time to JC polyomavirus (JCPyV) [1,2], and these were the only human polyomaviruses to be isolated until 2007. Since then 11 other human polyomaviruses have been discovered, including Merkel cell polyomavirus, thought to be the major cause of Merkel cell carcinoma, rare but aggressive tumours [3,4]. Their oncogenic potential alongside the discovery of many new polyomaviruses has led to renewed interest in these viruses, and has resulted in a revision of the polyomavirus taxonomy. Polyomaviruses are split into three genera: orthopolyomavirus (characterized by SV40 and includes BKPyV and JCPyV), wukipolyomavirus (characterized by KIPyV and WUPyV human polyomaviruses) and avipolyomaviruses (containing the avian polyomaviruses) [5]. BKPyV and JCPyV are still the only human polyomaviruses with reliable cell culture systems available for research, and thus are used alongside SV40 and murine polyomavirus as the main tools for studying the viral life cycle of this family of small non-enveloped DNA viruses.

The seroprevalance of polyomaviruses within the human population is relatively high, with BKPyV estimated to be present in up to 90% of the population [6–9]. Exposure generally occurs in childhood and is thought to result in a lifelong infection, with BKPyV and JCPyV most probably persisting in the kidneys at low levels [10–12]. There is no disease association of BKPyV in healthy individuals but the virus causes problems under circumstances of prolonged immune suppression. For example, BKPyV can re-activate in kidney transplant patients after immunosuppression, leading to polyomavirus-associated nephropathy (PVAN). PVAN occurs in 1–10% of kidney transplant patients, of which up to 80% develop graft rejection [13]. Reactivation can also lead to haemorrhagic cystitis (HC) in haematopoietic stem cell transplant patients [14]. The drugs most frequently used to target BKPyV in PVAN and HC are cidofovir and leflunomide, which, while being effective for HC [15], are nephrotoxic and therefore are not ideal for treatment of PVAN. This has resulted in reduction of immunosuppression upon detection of viraemia being the main means of controlling infection in renal transplant patients, which is far from ideal [16,17]. While infection of BKPyV causes cancer in animal models, there is no conclusive evidence to date that the same is true in humans [18].

Polyomaviruses have a relatively simple structure, consisting of a capsid containing a DNA genome of around 5 kb in length. The circular genome is composed of three parts, the non-coding control region containing the origin of replication, the early genes and the late genes [19]. The early gene region encodes large T-antigen (TAg) and small T-antigen (tAg), expressed from the same transcript via alternative splicing. Many polyomaviruses have also been shown to encode other alternatively spliced T-antigens [20,21]. The late region consists of the major capsid protein VP1, as well as the two minor capsid proteins VP2 and VP3. VP1 forms 72 pentamers, with one copy of either VP2 or VP3 located on the inside of each pentamer. The orthopolyomaviruses also encode agnoprotein, a small hydrophobic multifunctional protein [6,7,22].

While many studies have investigated the mechanism of entry and assembly of BKPyV, little is known about the release of newly formed virions from infected cells. While it is generally assumed that non-enveloped viruses are released through passive means such as cell lysis, recent evidence for a range of non-enveloped viruses suggests this may not always be the case. Poliovirus has been shown to induce the formation of autophagosome-like vesicles that are involved in viral egress [23]. The egress of the parvovirus Minute Virus of Mice (MVM) has been demonstrated to be through lysosomal or late endosomal vesicles [24]. An ESCRT (endosomal sorting complex required for transport)-dependent exosome-like pathway has also been identified as being involved in hepatitis A egress, leading to the release of hepatitis A virions contained within host-derived membranes [25]. Evidence has also been published suggesting SV40 release can occur before cell lysis, and furthermore SV40 release can be inhibited by monensin, an ionophore that inhibits secretory pathways [26].

The role of pH and ion homeostasis in cellular secretion is known to be important, with ion balance across membranes playing a crucial role. Anion permeability across cellular membranes is controlled by channels, protein pores that allow the transport of negatively charged ions across membranes. In contrast to cation channels, which are highly specific, anion channels are permeable to a range of different anions, the most abundant of which are chloride ions. Because of this anion channels are often named chloride channels despite their permissiveness to other anions such as sulfate, oxalate, bicarbonate, nitrate and other halides [27–29]. They have a range of functions and are particularly important in regulating pH in endosomes, lysosomes and the Golgi. There are five classes of chloride channels: cystic fibrosis trans-membrane conductance regulator, calcium-activated chloride channels, voltage-gated chloride channels (ClCs), ligand-gated chloride channels (GABA (*γ*-aminobutyric acid)- and glycine-activated) and volume-regulated chloride channels [28].

The role of chloride channels in cellular function can be addressed by using channel blocking inhibitors. A commonly used chloride channel inhibitor is 4,4′-diisothiocyano-2,2′-stilbenedisulfonic acid (DIDS), a disulfonic stilbene derivative. Stilbenes are a family of anion channel inhibitors that work by binding open channels and blocking the pore, either in a reversible or a non-reversible manner [29]. The chloride channels DIDS is known to inhibit are ClC-2, ClC-Ka, ClC-Kb, ClC-6, ClC-7, calcium-activated chloride channel, maxi-chloride channel, volume-regulated chloride channels and Band 3, a bicarbonate/chloride ion exchanger [28,30–32]. ClC-3, 4 and 5 are all considered to be insensitive to DIDS [33,34]. ClC-Ka and Kb are plasma membrane chloride channels, mainly located in the kidney, while ClC-6 and 7 are thought to be intracellular Cl^−^/H^+^ antiporters. DIDS is also capable of blocking the Golgi pH regulator (GPHR), which regulates Golgi acidification and is important for secretion [35]. As well as blocking chloride channels, stilbenes such as DIDS can inhibit anion exchange channels such as the Na^+^-dependent Cl^−^/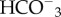-exchanger, which has a large effect on intracellular pH [36–38], as well as channels such as the glutamate transporter [39]. Recent evidence suggests DIDS may block vesicular exocytosis and preserve membrane integrity in neurons, and potentially other primary cells and tissues [40].

We have investigated the potential for non-lytic release of BKPyV, and the importance of anion homeostasis in BKPyV replication and release. Our data provide evidence of an active non-lytic release pathway for BKPyV that can be blocked by the anion channel inhibitor DIDS.

## 2. Results

### 2.1. Inhibition of anion channels decreases release of infectious BK

**Figure 1.**
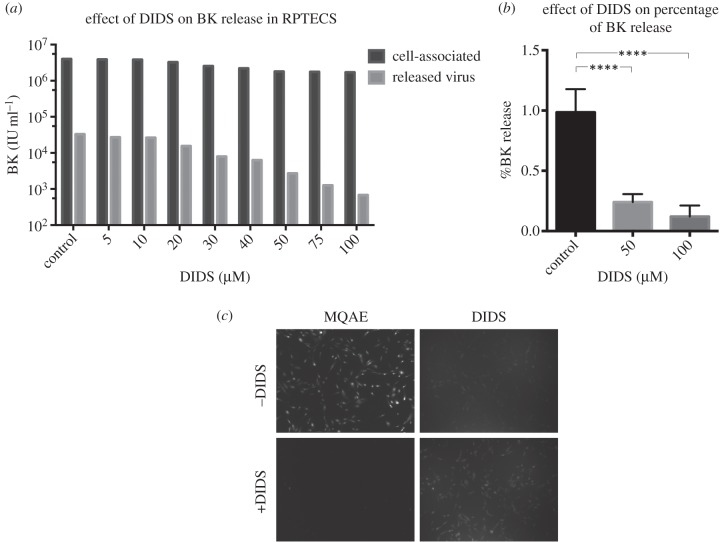
DIDS blocks release of infectious virions in the presence of DIDS. RPTE cells were infected at 1 IU cell^−1^ and treated with DIDS (or DMSO as a control) after 24 h. After 48 h, the media and cells were harvested separately. (*a*) A fluorescent focus assay was run to determine the IU ml^−1^ of BKPyV in the cells and supernatant. The percentage release was calculated under each condition and shown in table 1. The control, 50 and 100 µM DIDS were repeated four times, the percentage release calculated and the mean determined. (*b*) One-way ANOVA was run and the data found to be highly significant (*p* ≤ 0.0001). RPTE cells were treated with or without DIDS for 24 h, and MQAE added to cell for the last hour of incubation. (*c*) After washing with PBS, cells were imaged using a 10× lens on a wide-field fluorescence microscope.

The effect of DIDS on BKPyV release was tested in renal proximal tubule epithelial (RPTE) cells, a primary human cell line that supports efficient replication of BKPyV and the closest cell culture system to the natural site of infection for BKPyV currently available [41]. A range of concentrations of DIDS was used up to 100 µM. Cells were infected at 1 IU cell^−1^, DIDS added 24 h post-infection, and the supernatant and cells harvested separately at 48 h. BKPyV entry is thought to require 6–12 h before the genome reaches the nucleus [42] and new BKPyV progeny can be detected by 36–48 h. Therefore, this experimental set-up should avoid any potential effect of DIDS on virus entry, while having DIDS present throughout the assembly and egress of new virions. The viral titre was determined by fluorescent focus assay (figure 1*a*). The titre of the cell-associated virus reduced by no more than two-fold at any inhibitor concentration, while by 50 µM DIDS the released virus showed a 10-fold decrease. The percentage of virus released was 0.82% in the control, decreasing in a dose-dependent manner to 0.04% at 100 µM, a 20-fold reduction in viral release (table 1).

**Table 1.** Percentage release of BKPyV from RPTE cells treated with DIDS, calculated by dividing the supernatant viral titres and genome levels by cell-associated viral titres and genome levels from the data shown in figures 1 and 2.

		DIDS (µM)								
	control	5	10	20	25	30	40	50	75	100
infectious BKPyV	0.816	0.693	0.685	0.473	—	0.310	0.284	0.150	0.071	0.040
BKPyV genome	0.969	—	—	—	0.221	—	—	0.079	0.098	0.081

To determine the significance of the effect of DIDS on BKPyV release from cells, four independent experiments were performed with a DMSO control and DIDS added at 50 µM and 100 µm (figure 1*b*). The mean percentage release was calculated as 0.98%, 0.24% and 0.12% for the control, 50 and 100 µM DIDS, respectively, and one-way ANOVA was used to determine significance between the control and 50 or 100 µM DIDS treatment. Both were found to be significant at *p* ≤ 0.0001, where *p* ≤ 0.05 shows significance.

The effect of DIDS on BKPyV release was also tested at 72 h post-infection when greater total amounts of infectious virus are produced, with 50 µM DIDS present for the final 24 h of infection. These data showed a slightly higher overall release of virus from control cells by 72 h post-infection, at 2.1% of total infectivity, and that the presence of DIDS reduced virus release to 0.26%. Therefore, the presence of DIDS inhibits release of infectious BKPyV from RPTE cells at both early (48 h) and late (72 h) times post-infection.

In order to confirm the activity of DIDS as an inhibitor of chloride transport in these primary kidney epithelial cells, RPTE cells were incubated with or without 50 µM DIDS for 24 h and then a fluorescent indicator of intracellular chloride ions, MQAE (*N*-(ethoxycarbonylmethyl)-6-methoxyquinolinium bromide), was added for the last hour of incubation. Fluorescence microscopy imaging clearly demonstrated a strong signal for MQAE in control cells, but this was reduced to barely above background in the presence of DIDS, thus confirming the activity of this anion inhibitor in RPTE cells (figure 1*c*).

To investigate whether treatment of cells with DIDS for 24 h affects the viability of RPTE cells a trypan blue assay was used. The viability of cells was slightly decreased after adding DIDS; however, even at the highest dose of DIDS (100 µM) cell viability was 85% (electronic supplementary material, figure S1a). This may partially contribute to the small decrease in cell-associated virus, but cannot account for the large reduction in release of BKPyV caused by DIDS.

### 2.2. The anion channel inhibitor DIDS blocks BKPyV particle release

**Figure 2.**
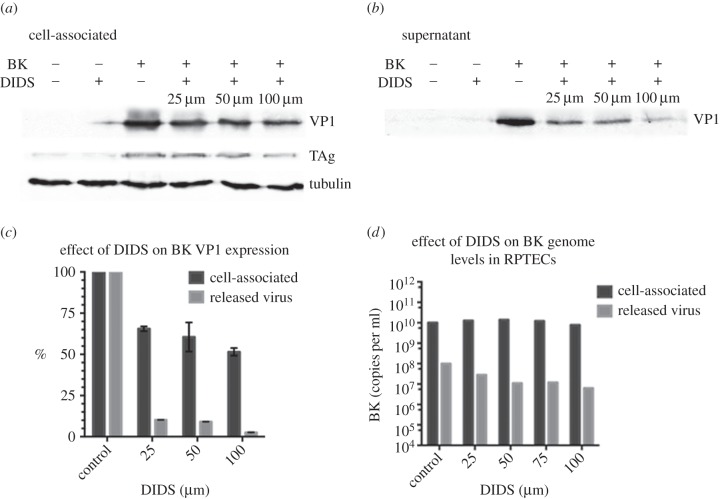
Analysis of released capsid proteins and viral genome support DIDS blocking viral release. (*a*–*b*) Effect of DIDS on viral capsid protein levels (*a*) in the cell and (*b*) released into the supernatant. RPTE cells were infected with BK-Dunlop at 1 IU cell^−1^ and had DIDS added 24 h post-infection. The virus was harvested at 48 h. The controls are uninfected and untreated RPTE cells; uninfected cells treated with 50 µM DIDS and infected cells treated with DMSO instead of DIDS. Tubulin was detected in the cell-associated sample as a loading control. (*c*) Quantification of the western blot data was performed using the Li-Cor Odyssey software on the VP1 bands present from the cell-associated virus and the virus released into the supernatant from two independent experiments. Error bars represent the distribution of the two datasets. (*d*) Genome levels were detected from the cell and supernatant samples using qPCR and analysed using Rotor-Gene.

The data shown above demonstrate that DIDS causes a reduction in the amount of infectious virus released from RPTE cells. However, this could be caused either by a reduction in the number of virions released or alternatively by equivalent release of particles but with the majority of virions being rendered non-infectious. To investigate this, RPTE cells were infected with BKPyV, treated with DIDS, and the cells and supernatant harvested separately. Ultracentrifugation was used to pellet the extracellular virus, and the protein content of both released and cell-associated samples were analysed by Western blot (figure 2*a,b*). The VP1 expression levels were quantified using the Odyssey infrared imaging system software by Li-Cor for two independent experiments (figure 2*c*). Viral DNA was also extracted from the infected cells as well as the supernatant samples and measured using qPCR (figure 2*d*). DIDS treatment caused at most a 50% reduction in the cell-associated levels of VP1, consistent with the infectivity assay data showing approximately a 50% decrease in cell-associated BKPyV titre. TAg expression decreased by similar amounts. The level of BKPyV genome within cells remained relatively unchanged, suggesting genome replication is unaffected by DIDS. Western blot analysis of the released virions demonstrated VP1 levels decreased to approximately 10% of the control at 25 µM, and to just 1% of the control from the cells treated with 100 µM DIDS. The percentage release of viral genomes also decreased in a dose-dependent manner from approximately 1% BKPyV release in control conditions down to 0.08% at 100 µM DIDS (table 1). The similar reductions observed for the release of virus protein and genomes compared with the reduction in infectious virus release in the presence of DIDS supports the hypothesis that DIDS blocks the release of virions from infected cells and is not simply reducing the infectivity of released BKPyV particles. To investigate whether changes in VP1 protein stability in the presence of DIDS could contribute to the slight decrease in cell-associated VP1 levels, or in the amount of VP1 detected in media samples, RPTE cells were infected with BKPyV, incubated with or without DIDS from 24 h post-infection and then treated with or without cycloheximide (CHX) to block translation. Cells were then harvested at 0, 12, 24 or 36 h after cycloheximide addition, and lysates examined by Western blot (electronic supplementary material, figure S1*b*). Normalization of VP1 levels to tubulin demonstrated the expected increase in VP1 levels in the absence of cycloheximide and decrease in the presence of cycloheximde, and importantly no difference was observed in the presence or absence of DIDS. These data suggest that there is no change in the expression or turnover rates of VP1 when cells are incubated with DIDS.

### 2.3. BKPyV localizes to acidic compartments within the cell after addition of DIDS

**Figure 3.**
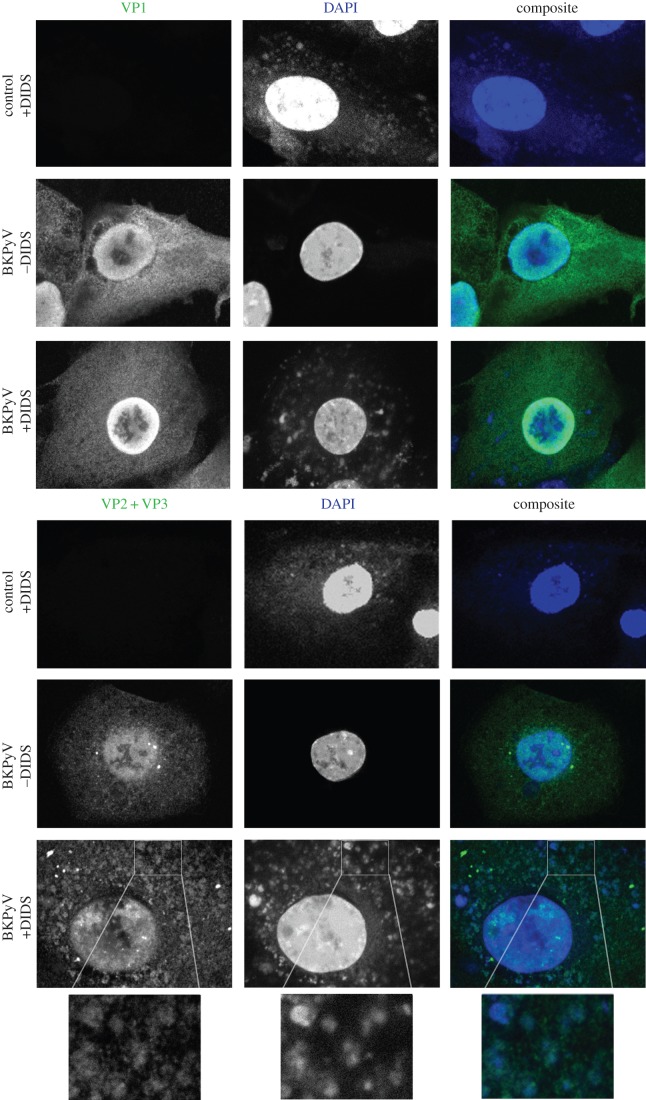
Effect on localization of viral proteins by DIDS. RPTE cells were left uninfected or infected at 1 IU cell^−1^ and were treated with DIDS or DMSO as a control after 24 h. Cells were fixed 48 h post-infection and stained for either VP1 (PAb597) or VP2/3 (red). DAPI staining is shown in blue, as is the auto-fluorescence of DIDS. Images are single z-slices acquired using confocal microscopy.

Immunofluorescence microscopy was used to determine whether incubation with DIDS resulted in a change of localization of the capsid proteins (figure 3). RPTE cells were infected at 1 IU cell^−1^, treated with 50 µM DIDS after 24 h and fixed at 48 h post-infection. Primary antibodies against VP1 and VP2/3 were used to determine whether any changes in viral protein localization occurred within the cell. TAg was also stained as a control and no changes in localization were observed (electronic supplementary material, figure S2). DIDS auto-fluoresces at 415 nm when excited by a maximum emission of 341 nm, and thus was detected alongside the nuclear stain DAPI [43], appearing as spherical compartments throughout the cytoplasm in RPTE cells. Cells infected with BKPyV did not appear to show any change in DIDS localization. The localization of VP2/3 in untreated cells was diffuse throughout the cell, with a stronger signal occurring in the nucleus. In the presence of DIDS, a change in localization occurred, with VP2/3 accumulating in spherical vacuole-like structures in the cytoplasm that co-localized with DIDS auto-fluorescence, suggesting a concentration of either BKPyV capsids or free VP2 or 3 in these structures. This VP2/3 staining was specific to infected cells and not the result of DIDS treatment alone. VP1 localized in a similar manner to VP2/3 in untreated cells, with strong nuclear expression but slightly less signal occurring in the cytoplasm. Surprisingly, no change in VP1 localization could be observed with cells treated with 50 µM DIDS. The same observations were made with two further independent VP1 antibodies (electronic supplementary material, figure S3), suggesting the observed vacuole-like structures in DID-treated cells either do not contain VP1, or that the three VP1-specific antibodies we have available do not recognize assembled BKPyV virions. To test this hypothesis, BKPyV particles were bound to the surface of RPTE cells by incubating on ice, followed by fixation and then incubation with each VP1-specific or the VP2/3-specific antibodies (electronic supplementary material, figure S4). These data demonstrate that the VP2/3 antibody efficiently recognized virions bound to the surface of cells, whereas none of the three VP1 antibodies demonstrated signals much above background. Therefore, the epitopes to which these VP1 antibodies bind appear to be inaccessible, whereas at least one epitope bound by the polyclonal anti-VP2/3 is accessible in fully formed virions, suggesting the VP2/3-positive vacuole-like structures observed in the presence of DIDS could contain mature virus particles.

**Figure 4.**
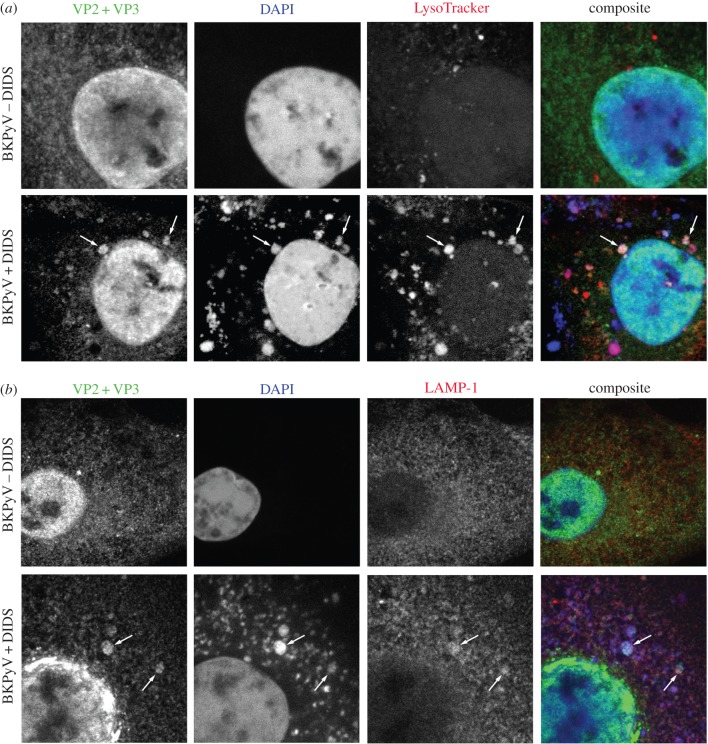
VP2 and 3 co-localize with LAMP-1 and LysoTracker in the presence of DIDS. RPTE cells were infected at 1 IU cell^−1^ and were treated with DIDS or DMSO as a control after 24 h. (*a*,*b*) Cells were fixed 48 h post-infection and stained for VP2 and VP3 (green) and LAMP-1 (*b*), or treated with LysoTracker 2 h before fixing (*a*) (red). DAPI staining is shown in blue, as is the auto-fluorescence of DIDS. Images are single z-slices acquired using confocal microscopy.

To determine the nature of the compartments where VP2/3 accumulates in the presence of DIDS, BKPyV-infected and DIDS-treated cells were stained with LysoTracker red, which marks acidic organelles with high selectivity (figure 4*a*), or probed with an antibody to the late endosomal/lysosomal marker LAMP-1 (figure 4*b*) alongside VP2/3. In untreated RPTE cells, LysoTracker red stained small puncta throughout the cytoplasm with occasional larger/brighter structures observed. In DIDS-treated cells, more numerous and larger LysoTracker red-stained structures were observed, which frequently co-localized with DIDS fluorescence. Furthermore, the VP2/3 containing vacuoles were both LysoTracker- and DIDS-positive. The increase in LysoTracker red staining was also observed in uninfected RPTE cells, suggesting that perturbing anion homestasis rather than BKPyV infection leads to an expansion of acidic compartments in these cells (electronic supplementary material, figure S5*a*). LAMP-1 staining in uninfected RPTE cells demonstrated a mainly diffuse localization and localized to small puncta that sometimes appeared more numerous in the perinuclear region. This was in contrast to distinct staining of perinuclear puncta that can be observed in other human cell lines such as HeLa, suggesting relatively low endogenous expression levels of LAMP-1 in RPTE cells (electronic supplementary material, figure S5*b*). In cells treated with DIDS, while the background signal for LAMP-1 was still high, there was an increase in LAMP-1 signal in the VP2/3 containing vacuoles. Taken together, these data suggest that BKPyV virions become trapped in acidic compartments of lysosomal or late endosomal origin upon DIDS treatment.

### 2.4. DIDS causes accumulation of BKPyV particles in large vacuolar structures in the cytoplasm

**Figure 5.**
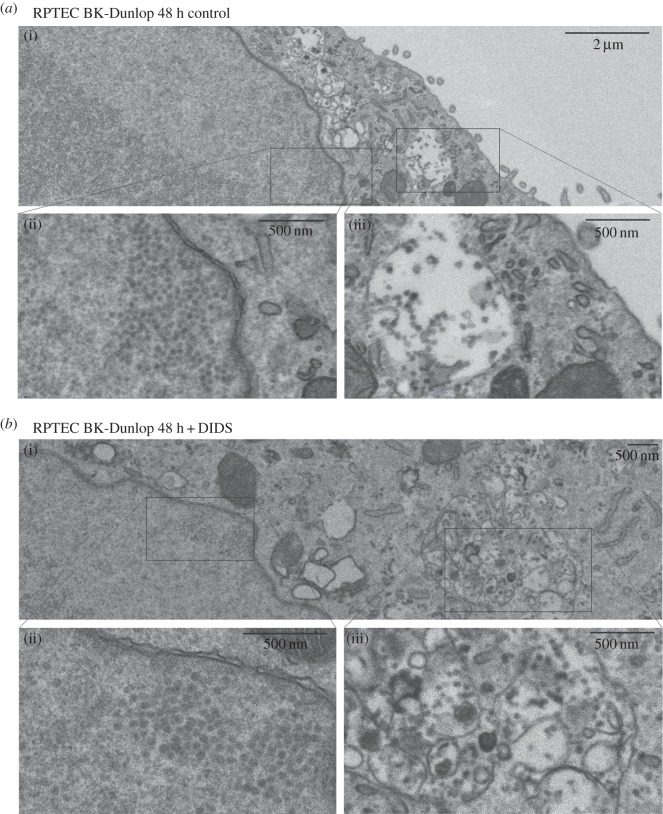
Transmission electron microscopy images of RPTE cells infected with BK-Dunlop and treated with DIDS. (*a*,*b*) RPTE cells were infected at 3 IU cell^−1^ for 48 h, with (*b*) DIDS or (*a*) a DMSO control added 24 h post-infection. (ii),(iii) Close ups from (i) in both conditions. (ii) Large numbers of BKPyV arranged in their nuclear factories and were used to determine whether the cell was infected. (iii) Compartments within the cytosol containing BKPyV particles (45 nm in size).

To investigate the effect of DIDS on infected RPTE cells at the ultra-structural level, transmission electron microscopy (TEM) was used (figure 5). Cells were infected at 3 IU cell^−1^ and fixed at 36, 48 or 60 h post-infection. DIDS (50 µM) or DMSO as a control was added to the cells 24 h prior to fixation. The BKPyV capsids have a diameter of approximately 45 nm and large arrays of capsids in the nucleus, often termed ‘virus factories' [44], were observed in infected cells (figure 5*a*,*b*(ii)). Virus particles were also observed in large cytoplasmic compartments resembling vacuoles, frequently with multiple membranous sections. These ranged in size from 500 nm^2^ to around 2000 nm^2^ and contained just a few up to several hundred virus particles. The addition of 50 µM DIDS had no visible effect on the nuclear arrays of virus; however, DIDS treatment caused a clear increase in the number of viral particles in the vacuolar compartments at all time points.

**Figure 6.**
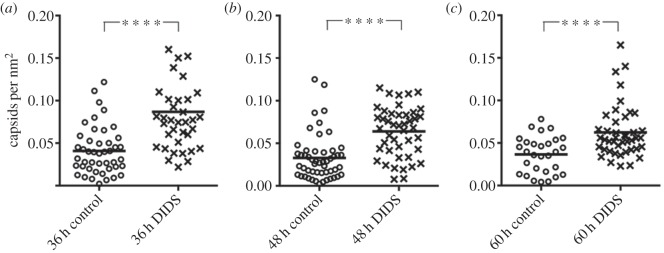
Quantification of TEM data. The number of BKPyV capsids counted in each compartment structure and calculated as number of capsids nm^−2^. RPTE cells were infected at 3 IU cell^−1^ and then fixed at 36 (*a*), 48 (*b*) or 60 h (*c*). A total of 50 µM DIDS or DMSO as a control was added 24 h before fixing. The Mann–Whitney test was used to determine significance, with values of *p* ≤ 0.0001 for all time points.

As the size of the compartments varied, to determine the approximate level of increase in BKPyV virions in cytoplasmic vacuoles upon DIDS treatment, the number of capsids per unit area (in square nanometre) was determined for each individual compartment using ImageJ software (figure 6). Only vacuole structures containing at least one BKPyV capsid were included. Over 30 virus-containing vacuoles from approximately 15 cells for each condition were quantified and the data presented as capsids per square nanometre. A significant increase in the number of capsids per nm^2^ in these compartments was observed in the presence of DIDS for all time points. These data suggest that inhibition of anion homeostasis by DIDS treatment causes the accumulation of BKPyV in large cytoplasmic vacuoles, consistent with the immunofluorescence data demonstrating increased VP2/3 localization in acidic lysosomal/late endosomal compartments upon DIDS treatment.

Taken together, our data demonstrate the presence of a non-lytic release pathway for BKPyV from infected RPTE cells that can be inhibited by disrupting cellular anion homeostasis. Furthermore, this non-lytic release pathway for BKPyV appears to involve acidic organelles with late endosomal or lysosomal characteristics.

## 3. Discussion

Polyomaviruses are becoming of increasing interest as our reliance on immunosuppressive therapies rises, and the discovery of new human polyomaviruses creates the possibility of these viruses being a significant risk factor for pathological conditions. The need to better understand polyomaviruses and to develop new therapeutic methods to treat them is of great importance. One area that is very poorly understood is the mechanism by which these viruses are released from infected cells. The dogma for non-enveloped viruses tends to be that they are simply released when infected cells undergo lysis, spilling infectious virus along with cytoplasmic contents into the extracellular environment. Release of viruses through cell lysis, either passively due to cytotoxic damage or actively via expression of lysis inducing viral proteins, would appear to be an inefficient mechanism that would be difficult to regulate for viruses to spread to new uninfected cells in a multicellular host. In particular, it may be advantageous for viruses that establish lifelong persistent infections, such as polyomaviruses, to adopt a more regulated non-lytic mechanism of virus release to reduce host inflammatory responses. Interestingly, evidence of non-lytic release mechanisms has recently been observed for non-enveloped positive strand RNA viruses (poliovirus and hepatitis A virus) and non-enveloped single-stranded DNA viruses (parvovirus) [23–25]. We now provide evidence for the existence of an active route of egress for BKPyV in primary renal cells that does not involve cell lysis. Our data demonstrate that approximately 1% of total infectious virus progeny is released into the media of cultured primary renal epithelial cells by 48 h post-infection and that this egress route can be inhibited by DIDS, an anion channel blocker known to effect cellular secretion pathways [32,35,40]. This suggests the presence of a specific and active route of BKPyV egress that does not involve cell lysis. As far as we are aware, this is the first evidence of non-lytic release for a human polyomavirus, and supports previous data from Clayson *et al*. [26] that suggested SV40 (a primate polyomavirus) can also be released prior to cell lysis.

Using a viral release assay, we were able to show a significant dose-dependent decrease in infectious BKPyV released into the cell culture media in the presence of DIDS, with only a small effect on viral titres within the cell. We confirmed this by measuring released virions by Western blotting of the major capsid protein (VP1) and qPCR detection of BKPyV genomes. Using all three methods, we observed a more than 10-fold reduction of BKPyV release from RPTE cells treated with DIDS, supporting the notion of a crucial role of anion homeostasis in non-lytic BKPyV egress. Furthermore, these data suggest that DIDS blocks the physical release of virions from infected cells, rather than simply rendering a proportion of released virions non-infectious.

To gain insight into the mechanism by which BKPyV is released in a non-lytic fashion, we analysed the localization of viral proteins in the presence or the absence of DIDS by immunofluorescence microscopy and virus particles by electron microscopy. These data demonstrated an increased accumulation of BKPyV virions in large cytoplasmic vacuoles in the presence of DIDS. Furthermore, accumulation of VP2/3 in LAMP-1-positive acidic organelles suggests that late endosomal/lysosomal compartments are involved in the pathway of non-lytic BKPyV release. Interestingly, we also observed a dramatic increase in the number and size of acidic compartments in DIDS-treated cells that was independent of BKPyV infection, and DIDS also accumulated in these compartments. This suggests that DIDS increases the acidity of certain subcellular compartments, presumably due to disrupting anion balance across membranes, which leads to an expansion of LAMP-1-positive trafficking compartments, where BKPyV particles become trapped.

Given that we observed a clear increase in BKPyV virions within cytoplasmic vacuoles by electron microscopy, it was surprising that we only detected an accumulation of the minor capsid proteins VP2/3 in acidic organelles but not the major capsid protein VP1. However, all three VP1-specific antibodies could not detect BKPyV particles bound to the surface of cells, suggesting the epitopes bound by these antibodies are inaccessible in mature virions, and presumably only bind to free VP1 in the nucleus and cytoplasm of infected cells. In the known polyomavirus structures, the minor capsid proteins face inward towards the viral genome within the virion, with one copy of VP2 or 3 on the inside of each VP1 pentamer, suggesting that VP2 and 3 would be predicted to be fairly inaccessible to antibody recognition. However, the VP2/3 antibody clear binds BKPyV particles bound to the surface of cells, suggesting at least one domain of VP2 or 3 is exposed enough in the virion structure to efficiently bind immunoglobulins.

At present, the cellular pathway that BKPyV uses to undergo non-lytic release from infected cells is still poorly characterized. This pathway is clearly inhibited by DIDS in a dose-dependent manner. As DIDS is a well-defined and commonly used anion channel inhibitor, it seems likely that the pathway of non-lytic BKPyV release is sensitive to anion homeostasis, perhaps relating to the control of organelle pH. Furthermore, the co-localization of LAMP-1 and LysoTracker with BKPyV capsid proteins and DIDS in cytoplasmic vacuoles suggests an involvement of late endocytic and/or lysosomal-related compartments in the non-lytic release of BKPyV release. Lysosomes and lysosome-related organelles have the capacity to be secretory organelles, with specialized cellular pathways that regulate their secretion present in various cell types [45]. Interestingly, DIDS has been shown to inhibit the lysosomal enzyme secretion from human neutrophils, suggesting a role of anion homeostasis in controlling the fusion of lysosome-related organelles with the plasma membrane [46]. It will be interesting to investigate whether any of the cellular trafficking mediators important for lysosome-related organelle secretion, such as Rab27a, are also involved in BKPyV release.

Regarding the anion channel(s) that are important for BKPyV release, one complication is the large number of channels and transporters present within cells, many of which are known to be DIDS-sensitive. The members of the anion channel family that are likely to be most relevant to BKPyV release are ClCs 3–7, which are widely expressed in mammalian cells and are predominately localized to intracellular compartments [47,48]. All five are Cl^−^/H^+^ antiporters [49] and play a critical role in pH regulation, although there is a slight variation in localization between them. ClC-6 and ClC-7 appear to be good candidates for further investigation—both these Cl^−^/H^+^ antiporters are sensitive to DIDS inhibition and are localized to late endosomes and/or lysosomes [50,51]. Furthermore, ClC-5, despite being regarded as relatively insensitive to DIDS, could warrant further investigation as this Cl^−^/H^+^ antiporter is highly expressed in kidney cells and has been shown to be localized to endosomes in kidney proximal tubule cells [52,53]. Interestingly, DIDS also inhibits the activity of the GPHR, an anion channel that has been shown to be important for cellular secretion pathways [35]. However, at present we cannot rule out the possibility that DIDS is interfering with BKPyV release indirectly, perhaps through inhibition of some cellular function related or even unrelated to anion homeostasis.

One surprising observation from this work was the increase in acidic cytoplasmic vacuoles (demonstrated by LysoTracker staining) upon DIDS treatment. This is somewhat counterintuitive because chloride channel/transporters have previously been shown to facilitate luminal acidification, most probably by providing the neutralizing current for the increase in positive charge that would otherwise occur in the lumen of acidic organelles due to the proton-pumping activity of V-type H^+^-ATPases (reviewed in [47]). Indeed, this has been demonstrated for both ClC-3 and ClC-5, where endosomal pH became more alkaline in cells from the respective ClC knockout mice [53,54]. However, it has also been demonstrated that lysosomal pH is unaffected in mice lacking either ClC-6 or ClC-7 [55,56]. The relationship between anion homeostasis and late endosome/lysosome pH is currently unclear, although our data suggest inhibition of DIDS-sensitive channels causes an increase in the size of certain acidic compartments, most probably lysosome-related, within RPTE cells.

An important area for further work will be determining how BKPyV virions gain access to the lumens of late endosome/lysosome-related cytoplasmic compartments during non-lytic egress. Despite their relatively small size of approximately 45 nm diameter, it is difficult to envisage how BKPyV virions could be transported from the cytoplasm into the lumen of cytoplasmic compartments without membrane budding, such as occurs during the formation of exosomes and the intra-luminal vesicles (ILVs) in multivesicular endosomes. Interestingly, the cellular machinery responsible for mediating exosome and ILV formation, the ESCRT pathway, has recently been implicated in the release of hepatitis A viruses in a process which leads to the normally non-enveloped hepatitis A virions being contained within host-derived membranes [25]. In the future, it will be interesting to investigate the potential involvement of the ESCRT pathway in non-lytic BKPyV release, and to establish whether released BKPyV virions are associated with membranes. One other potential route for non-lytic BKPyV egress could be similar to the autophagosome-mediated exit without lysis pathway proposed for poliovirus [23].

In summary, our data show clearly that BKPyV has an active and non-lytic method of egress from infected cells, and does not rely solely on cell lysis. We propose that such a mechanism may help polyomaviruses to establish lifelong persistent infections in their natural hosts, by enabling low-level dissemination of infectious virus to uninfected cells without the need to cause cell lysis and resulting inflammation and tissue damage. These findings open up several new areas for investigation on this important family of human and animal pathogens.

## 4. Material and methods

### 4.1. Virus, cell lines and antibodies

RPTE cells were obtained from Lonza and used at passage 6 and 7 for all experiments. RPTE cells were grown in renal epithelial basal media supplemented with the REGM bulletkit (Lonza).

BK-Dunlop inserted into a pGEM vector (kindly provided by M. Imperiale) was digested using BamHI, then purified and re-ligated. The re-ligated genome was transfected into a T75 flask of RPTE cells in 5% FCS REGM. After three weeks, the cells were split into three T150 flasks and left for a further three weeks before harvesting. The cells were freeze thawed three times to release the virus and assayed using a fluorescent focus assay (protocols based on [57]).

The primary antibodies used were PAb597 against SV40 VP1 (kindly provided by W. Atwood), P5G6 against BKPyV VP1 (kindly provided by D. Galloway), ab53977 against SV40 VP1 (Abcam), ab53983 against SV40 VP2 and VP3 (Abcam), PAb416 against SV40 T-antigen (Abcam), H4A3 against LAMP-1 (Developmental Studies Hybridoma Bank) and ab6160 against tubulin [YL1/2] (Abcam). Secondary antibodies used were Alexa Fluor 568 donkey anti-mouse and Alexa Fluor 488 or 568 goat anti-rabbit (Invitrogen). LysoTracker red DND-99 was obtained from Life Technologies.

### 4.2. Cell infections and harvesting virus

For viral release assays, RPTE cells were infected with BK-Dunlop at 1 IU cell^−1^. After 1 h, the medium was removed, the cells gently washed in PBS and then fresh medium added. Twenty-four hours post-infection DIDS was added. DIDS was obtained from Life technologies and made up at 100 mM in DMSO. Controls used an equivalent volume of DMSO as the highest concentration of DIDS used. Forty-eight hours post-infection the virus was harvested. The supernatant was removed and centrifuged for 5 min at 2000*g* to pellet any cell debris in the media, and then the supernatant transferred to new tubes. This was repeated to ensure no cell debris was present before centrifuging at 100 000*g* for 2 h to pellet the virus. The media was aspirated and either resuspended to be assayed using immunofluorescence and qPCR or left as a pellet for Western blots. The RPTE cell monolayer was harvested separately in 1 ml of REGM.

### 4.3. Fluorescent focus unit assay and immunofluorescence

For the fluorescent focus unit (FFU) assay, RPTE cells were grown on coverslips and infected with serial dilutions of cell-associated virus or released virus. After 48 h, the cells were fixed in 3% formaldehyde, permeabilized and quenched (50 mM NH_4_Cl and 0.1% Triton X-100 in PBS), blocked in PGAT (0.2% gelatin, 0.01% triton, 0.02% NaN_3_ in PBS) and stained using pAB597. Slow fade gold mounting reagent with DAPI (Invitrogen) was used to mount the coverslips and stain the nuclei. All conditions were performed in duplicate and the numbers of infected cells were counted in five fields of view from each of the duplicates. The IU ml^−1^ was determined by calculating the number of infected cells in the entire well from the mean number of infected cells in the 10 fields of view, and then the number of infectious units calculated. For immunofluorescence, RPTE cells were infected at 1 IU cell^−1^ and 50 µM DIDS added after 24 h. Forty-eight hours post-infection, the cells were fixed, blocked and permeabilized. For cells treated with LysoTracker, medium was removed 2 h before fixing and fresh medium containing LysoTracker red was added. Primary antibodies used were P5G6, pAB597, ab53977 (VP1), ab53983 (VP2/3) and ab25630 (LAMP-1) and the secondary antibody used were Alexa Fluor 568 donkey anti-mouse or goat anti-rabbit and Alexa Fluor 488 donkey anti-rabbit. Coverslips were mounted with slow fade gold containing DAPI. Cells were imaged using a 63× oil immersion lens on a Leica SP5 confocal microscope or an Olympus IX81 wide-field fluorescence microscope.

### 4.4. Intracellular chloride ion assay

RPTE cells were treated with 50 µM DIDS or an equal volume DMSO as a negative control for 23 h, followed by incubation with 5 mM MQAE for 1 h. The cells were washed five times with PBS and imaged using the Olympus IX70 fluorescence microscope with a 10× objective.

### 4.5. Cell viability assay

The viability of RPTE cells was determined using a trypan blue assay. Cells were grown for 24 h before adding DIDS and DMSO of an equal volume to the highest DIDS concentration was used as a control. Twenty-four hours after adding the inhibitor, the cells were detached using trypsin/EDTA and incubated with 0.4% trypan blue (Sigma) for 2 min before determining the percentage of cells that had taken up the dye using a hemocytometer. All conditions were performed in duplicate.

### 4.6. Western blot and quantification

Cell lysate preparation and Western blots were performed as described in [58]. Primary antibodies used were P5G6, PAB416 and ab6160 as a loading control (VP1, TAg and tubulin, respectively) followed by IRDye680-, or IRDye800-conjugated secondary antibodies. Imaging was performed using the Li-Cor Odyssey Infrared Imaging system. Quantification was performed using the Odyssey software.

### 4.7. VP1 stability assay

RPTE cells were infected at 1 IU cell^−1^ and treated with or without 50 µM DIDS at 24 h post-infection in the presence or the absence of 50 µg ml^−1^ cycloheximide. Cells were then harvested at 0, 12, 24 or 36 h after cycloheximide addition, and lysates examined by Western blot with antibody P5G6 and ab6160 (VP1 and tubulin, respectively).

### 4.8. Real-time PCR (qPCR)

RPTE cells were infected with BKPyV at 1 IU cell^−1^, treated with DIDS at 24 h and harvested at 48 h. The cell and supernatant virus samples were lysed using 10% SDS and 20 mg ml^−1^ proteinase K in TE buffer before performing a phenol/chloroform extraction. Primers and the probe for qPCR were designed and obtained through TIB MOLBIOL and target a region in the agnoprotein gene (forward primer: TGTCACGWMARGCTTCWGTGAAAGTT; reverse primer: AGAGTCTTTTACAGCAGGTAAAGCAG; Taqman probe: 6FAM-TTTTGCTGGAMTTTTGYASAGGTGAAGACAGTGT—BBQ). The qPCR reaction used 300 nM of each primer and 50 nM Taqman probe, run using a Rotor-Gene (RG-3000, Corbett Research) and analysed using the Rotor-Gene software.

### 4.9. Detection of BKPyV virions bound to cells

RPTE cells were chilled on ice, and then infected with BKPyV at 1 IU cell^−1^ for 1 h on ice. Cells were then fixed with ice-cold formaldehyde, blocked with PBS + 2% fetal calf serum and then incubated with primary antibodies to VP1 or VP2/3. Cells were washed, incubated with Alexa Fluor-568 conjugated secondary antibodies, washed and then coverslips were mounted with slow fade gold containing DAPI. Cells were imaged using a 63× oil immersion lens on an Olympus IX81 wide-field fluorescence microscope.

### 4.10. Transmission electron microscopy and quantification

RPTE cells were infected at 0.5 IU ml^−1^ and incubated for 36, 40 and 60 h. DIDS was added to cells at 50 µM 24 h before fixing. The cells were washed with 0.9% (w/v) sodium chloride and fixed in 2% glutaraldehyde/2% formaldehyde in 0.5 M sodium cacodylate buffer (pH7.4) for 4 h at 4°C. After fixing, the cells were scraped up, pelleted and washed in buffer to remove fixative. The cell pellets were treated with 1% osmium ferricyanide for 2 h, before being washed in distilled water and treated with 2% uranyl acetate in 0.05 M maleate buffer (pH 5.5) for 2 h. The samples were rinsed again in distilled water before being dehydrated in graded ethanol, treated with dry acetonitrile and infiltrated with Quetol epoxy resin. Ultrathin sections were examined with an FEI Technai G2 Transmission electron microscope operated at 120 Kv using an AMT XR60B digital camera running Deben software. Samples were processed by the Cambridge Advanced Imaging Centre, University of Cambridge. Determining the number of capsids per nm^2^ was performed using ImageJ software. The area of each compartment was measured and the number of capsid structures present counted.

## Supplementary Material

Supplementary Data legends revised.docx

## Supplementary Material

Figure S1. Effect of DIDS on RPTE cell viability and VP1 expression levels.

## Supplementary Material

Figure S2. Effect of DIDS on localisation of TAg.

## Supplementary Material

Figure S3. Investigating the effect of DIDS on localisation of VP1 using additonal antibodies.

## Supplementary Material

Figure S4. Assessing the detection of intact BKPyV virions by the available VP1 and VP2/3 antibodies.

## Supplementary Material

Figure S5. Lysotracker and LAMP-1 antibody staining in uninfected cells.
