# Supplementary material for: Anion homeostasis is important for non-lytic release of BK polyomavirus from infected cells
Source: Open Biol. 2015 Aug 5;5(8):150041. doi: 10.1098/rsob.150041 (PMC4554916; doi:10.1098/rsob.150041)
Supplement: Supplementary Data legends revised.docx [file rsob150041supp1.docx]

# Supplementary Data Legends

**Figure S1. Effect of DIDS on RPTE cell viability and VP1 expression levels.** (a) RPTE cells were treated with DIDS for 24 hours and their viability determined using a trypan blue assay (Sigma). Viability decreased to a maximum of 85% at 100μM. (b) RPTE cells were infected at 1 IU/cell and were then treated with DIDS or DMSO as a control, with or without cycloheximde. Cells were harvested at 0, 12, 24 and 36 hours after cycloheximide addition and analysed by western blot.

**Figure S2. Effect of DIDS on localisation of TAg.** RPTE cells were infected at 1 IU/cell and were treated with DIDS or DMSO as a control after 24 hours. Cells were fixed 48 hours post-infection and stained for TAg (red). DAPI staining is shown in blue, as is the auto-fluorescence of DIDS.

**Figure S3. Investigating the effect of DIDS on localisation of VP1 using additonal antibodies.** RPTE cells were infected at 1 IU/cell and were treated with DIDS or DMSO as a control after 24 hours. Cells were fixed 48 hours post-infection and stained using alternative VP1-specific antibodies P5G6 or ab53977 (red). DAPI staining is shown in blue, as is the auto-fluorescence of DIDS.

**Figure S4. Assessing the detection of intact BKPyV virions by the available VP1 and VP2/3 antibodies.** RPTE cells were incubated with 1 IU/cell on ice for 1 hour and then fixed and stained with the indicated antibodies (red). DAPI staining is shown in blue.

**Figure S5. Lysotracker and LAMP-1 antibody staining in uninfected cells.** (a) RPTE cells were treated with DIDS for 24 hour, and incubated with Lysotracker red for the last 2 hours prior to fixation. DAPI staining is shown in blue, as is the auto-fluorescence of DIDS. (b) RPTE cells and HeLa cells were fixed and stained with a LAMP-1 specific antibody.
