# Supplementary figures and images for: Anion homeostasis is important for non-lytic release of BK polyomavirus from infected cells
Source: Open Biol. 2015 Aug 5;5(8):150041. doi: 10.1098/rsob.150041 (PMC4554916; doi:10.1098/rsob.150041)

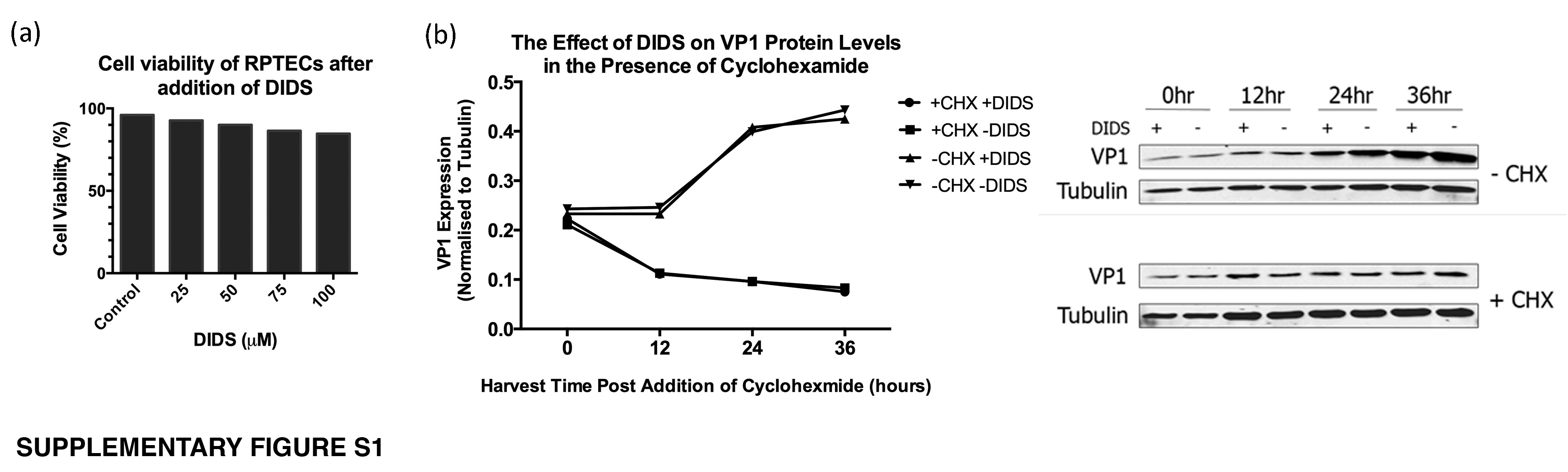

Supplement: Figure S1. Effect of DIDS on RPTE cell viability and VP1 expression levels. [file rsob150041supp2.jpg]

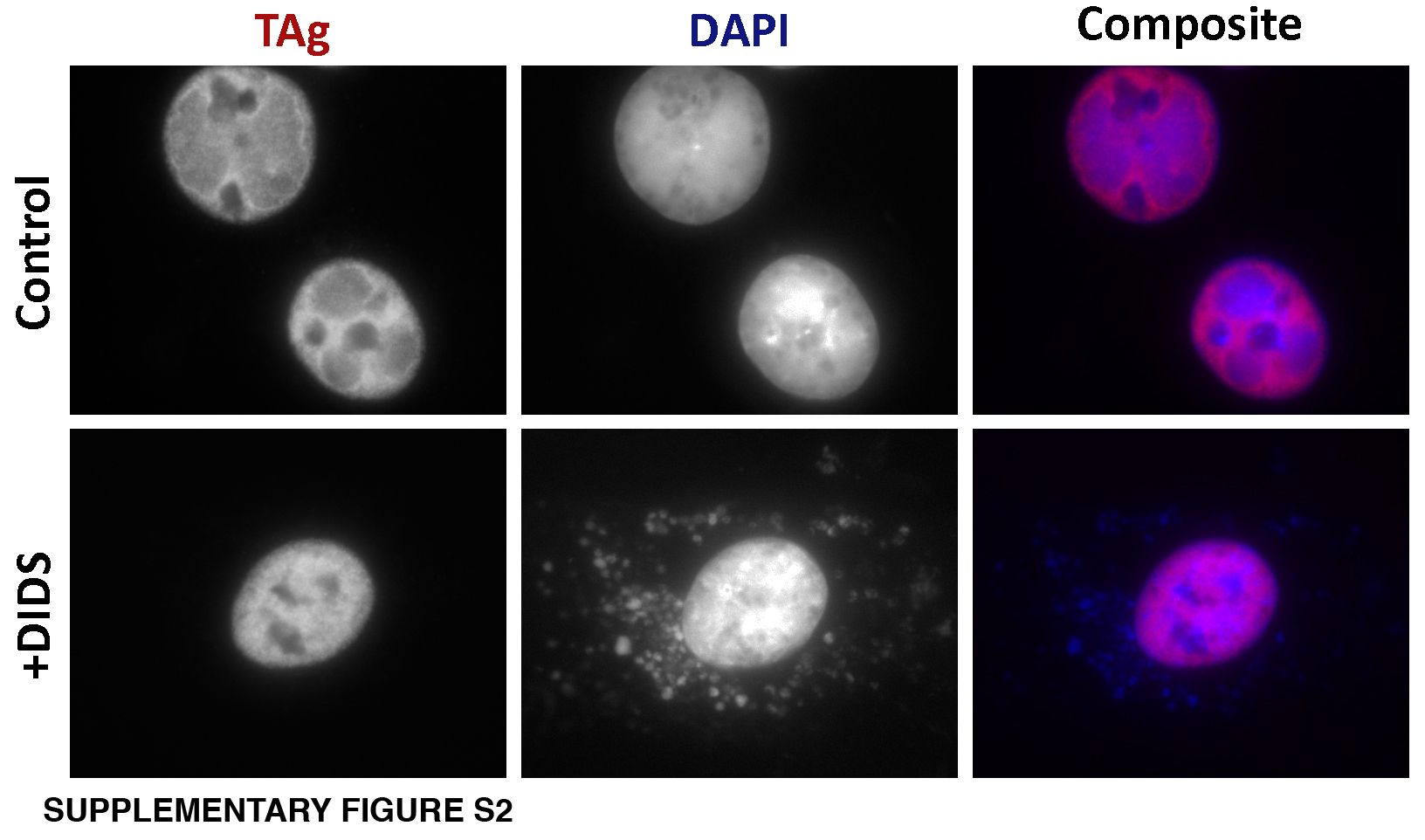

Supplement: Figure S2. Effect of DIDS on localisation of TAg. [file rsob150041supp3.jpg]

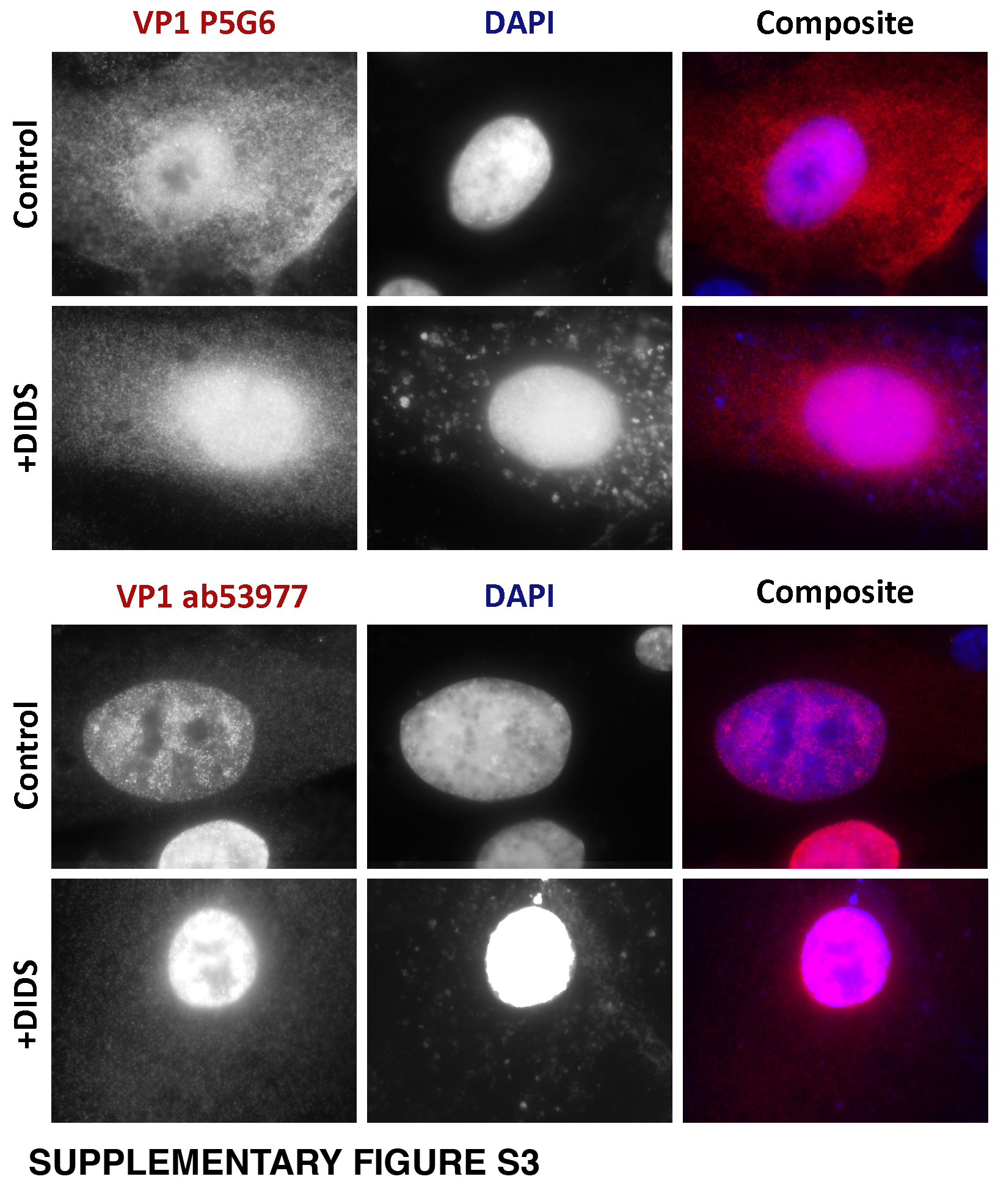

Supplement: Figure S3. Investigating the effect of DIDS on localisation of VP1 using additonal antibodies. [file rsob150041supp4.jpg]

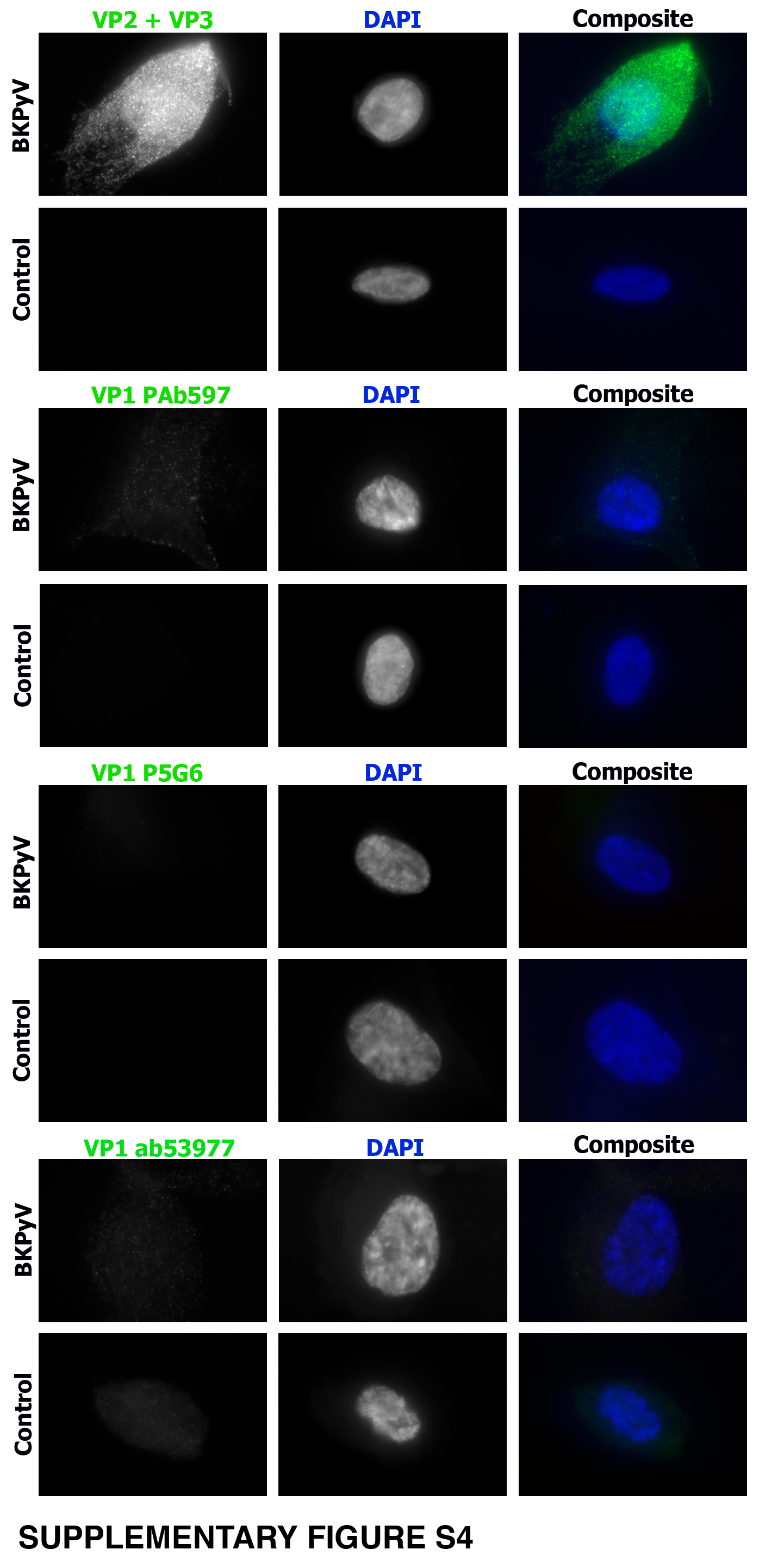

Supplement: Figure S4. Assessing the detection of intact BKPyV virions by the available VP1 and VP2/3 antibodies. [file rsob150041supp5.tif]

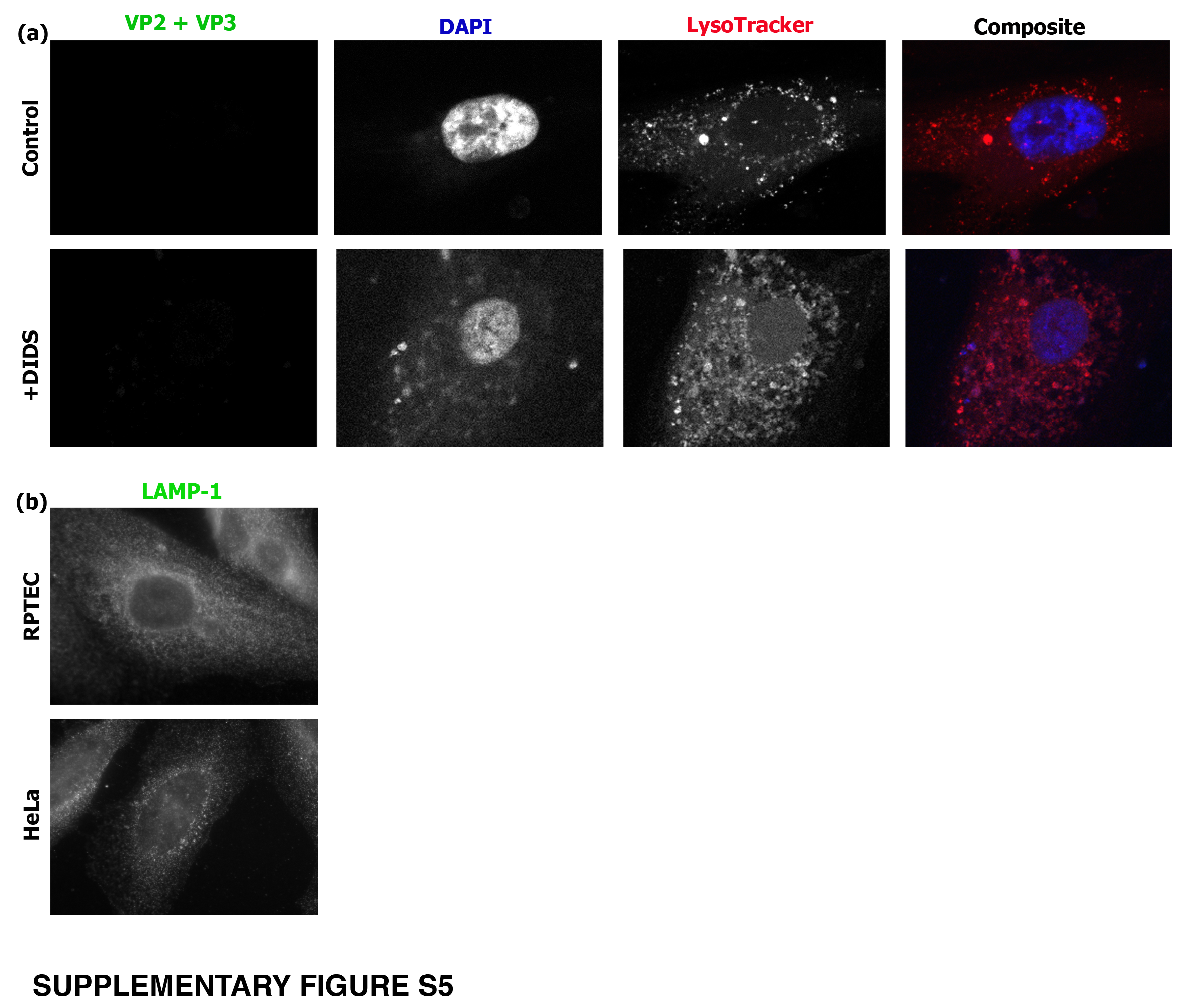

Supplement: Figure S5. Lysotracker and LAMP-1 antibody staining in uninfected cells. [file rsob150041supp6.tif]
